# Speeded-Up Focus Control of Electrically Tunable Lens by Sparse Optimization

**DOI:** 10.1038/s41598-019-48900-z

**Published:** 2019-08-26

**Authors:** Daisuke Iwai, Hidetoshi Izawa, Kenji Kashima, Tatsuyuki Ueda, Kosuke Sato

**Affiliations:** 1 Osaka University, Graduate School of Engineering Science, Toyonaka, 560-8531 Japan; 2 Kyoto University, Graduate School of Informatics, Kyoto, 606-8501 Japan

**Keywords:** Applied mathematics, Imaging and sensing

## Abstract

Electrically tunable lenses (ETL), also known as liquid lenses, can be focused at various distances by changing the electric signal applied on the lens. ETLs require no mechanical structures, and therefore, provide a more compact and inexpensive focus control than conventional computerized translation stages. They have been exploited in a wide range of imaging and display systems and enabled novel applications for the last several years. However, the optical fluid in the ETL is rippled after the actuation, which physically limits the response time and significantly hampers the applicability range. To alleviate this problem, we apply a sparse optimization framework that optimizes the temporal pattern of the electrical signal input to the ETL. In verification experiments, the proposed method accelerated the convergence of the focal length to the target patterns. In particular, it converged the optical power to the target at twice the speed of the simply determined input signal, and increased the quality of the captured image during multi-focal imaging.

## Introduction

Electrically tunable lenses (ETL), also known as liquid lenses, allow variable-distance focusing of imaging and display systems without mechanical structures. ETLs are focused by changing the electric signal applied on the lens, providing a more compact and inexpensive focus control than conventional computerized translation stages. Currently, ETLs can be realized by electro-wetting, liquid crystals, and polymer membranes^1^. Electro-wetting lens has the characteristics of large power range and fast response speed, while it requires high driving voltage and the aperture size is limited. Liquid crystal lens achieves larger focal length changes with lower applied voltage, though it suffers from chromatic aberration. Polymer-based liquid lenses allow the flexible selection of aperture size^2^. The present paper focuses on polymer-based ETLs, hereafter simply referred to as ETLs. The core forming the lens contains an optical fluid, which is sealed off by an elastic polymer membrane. An electromagnetic actuator ring exerts pressure on the outer zone of the container, which changes the curvature of the lens. The optical power of the lens is controlled by changing the electrical current flowing through the actuator coil. Polymer-based ETLs achieve a faster focal change than other ETL types, while maintaining a relatively large aperture size. Consequently, they have been exploited in a wide range of optical systems, from micro-scale systems such as microscopes (e.g., two-photon microscopy^3–6^, light-sheet microscopy^7,8^, selective-plane illumination microscopy^9,10^, confocal microscopy^11^, photoacoustic mictoscopy^12^, and phase microscopy^13^), optical coherence tomography^14^, optical diffraction tomography^15^, and laparoscopes^16^, to larger-scale systems such as still cameras^17^, video cameras^18^, depth cameras^19^, projectors^20^, video see-through head-mounted displays (HMDs)^21–23^, and optical see-through HMDs^24,25^.

In most of these systems, focal sweep of the ETL is applied in focal stacking, multi-focal, and extended depth-of-field (DOF) imaging and display^3–7,9,11–13,15,17,18,20,22–25^. The sweep patterns vary from a unit step function to various periodic ones (e.g., sinusoidal, rectangular, triangle, and staircase waves). However, although ETLs achieve fast focal change, their response time is limited by rippling of the optical fluid after actuation. For example, the rise time of an ETL (Optotune EL-10-30) by an input signal of a unit step function is 2–4 ms (where the rise time is the time taken by the ETL’s focal length to change from 10 to 90% of the range between the original and target focal lengths). Meanwhile, the lens resonates at high frequency (150 and 600 Hz), and fully settles only after 15 ms. Therefore, when the electric signal of the sweep patterns contains resonant frequency components of the lens (e.g., rectangular and staircase functions), the same waveform is not accurately replicated in the focal length. In past studies, the settling time of a step response has been reduced by applying low-pass filters, adding overshoot, or multiplying the original signal by a correction factor^7,8,24^. However, all of these approaches require manual trial-and-error parameter adjustments to achieve the desired focal length response. Moreover, the parameters need to be readjusted after every change of the sweep pattern or ETL aperture diameter.

This paper presents a computational method that optimizes the temporal pattern of the electrical signal fed to an ETL, speeding the convergence of the focal length to the target pattern. The major contribution is applying a sparse optimization framework to the computation of the input signal to minimize the differences between the output and target temporal patterns of the focal length. We regard the ETL as a linear time-invariant (LTI) system with static output nonlinearity, which is justified through experimental validation shown in Modeling and optimization section. By the definition of LTI, we can estimate the optical power of the ETL (i.e., the inverse of the focal length) simply by convolving the input signal with the system’s impulse response. The optimal input signal is then computed by minimizing the residual error between the temporal patterns of the estimated and target optical powers. In particular, the *L*_1_-norm penalty of the residual error is imposed in order to enhance its sparsity, i.e., to maximize the time duration over which the output signal is equal (or almost equal, practically) to the target signal (even in the continuous-time setting^26,27^), while accepting a relatively small number of large errors (outliers). This strategy is more useful than minimizing the total error by the least-squares method, particularly for a steeply rising target pattern with a relatively long plateau (such as a staircase function), because it more quickly converges the output on the target plateau despite the large pre-convergence errors and most modern imaging (display) systems can open the shutter (turn on the light source) only at convergence. Besides the error term, we add a smoothing term in the optimization framework which minimizes the second-order differential of the input signal, maintaining it temporally smooth. *L*_1_ constraint sometimes allows large outliers, which means the input signal may have impulse-like behavior. To prevent this undesirable situation, we add *L*_2_ penalty to guarantee its smoothness and suppress of outliers. In summary, for the optimal input signal, our method minimizes the weighted sum of the error term and the smoothing term. The convergence speed of the proposed method is evaluated in experiments.

## Results

Two experiments were conducted in prototype systems (see Experimental Setup in the Methods section). We first investigated the acceleration of the convergence of the ETL’s optical power by the proposed method with unit step patterns as targets. Assuming imaging application scenarios, we then investigated the image qualities captured by a camera with an ETL in a multi-focal imaging experiment, where capturing targets were placed at three different distances from the ETL. The optical power of the ETL was measured by a photodiode (see Experimental Setup in Methods section).

### Focus control for unit step patterns

In the first experiment, we measured the convergence speed of focus control by the proposed method for simple target patterns (unit step functions) of the ETL’s optical power. To be more precise, we take three electric currents to be injected to the ETL; 70, 130, and 190 mA, for which the corresponding optical powers are denoted as *D*_*c*70_, *D*_*c*130_ and *D*_*c*190_. Then, two of the currents were paired as the initial and final values of each step, giving six target step patterns in total. The input electric current for each target step pattern was determined by eight methods, one naïve and seven proposed methods, and their outputs were compared. In the naïve method, the input electric currents were unit step signals formed from pairs of 70, 130, or 190 mA corresponding to the various target patterns. In the proposed methods, the input signal was computed to minimize the weighted sum of the residual error and the smoothing terms, as described in the Introduction. To investigate the effect of the weight applied to the smoothing term, we prepared seven weight values (0.01, 0.1, 1, 10, 100, 1000, and 10000), giving the seven proposed methods. Overall, we prepared 48 experimental conditions (6 step patterns × 8 methods), and compared the settling times in the proposed and naïve methods. The settling time is defined as the period from when the optical power exceeds the initial range to when it converges within the final range. The initial (final) range is ±10 % of the step height from the initial (final) value of the target step pattern.

**Figure 1:**
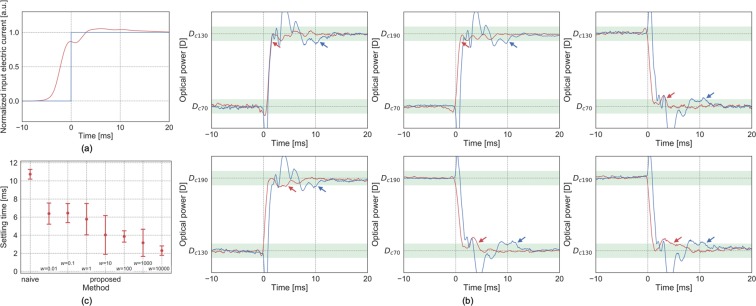
Focus control results for unit step patterns. (**a**) Normalized input signal and (**b**) optical powers for the six target patterns with a weight of 1000 (blue: naïve, red: proposed, arrows: settling time, green area: ±10% from the initial and final values of the target step pattern). (**c**) Average and standard deviation of the settling time in each experimental method.

We computed the input signals for the above-mentioned 48 conditions. Because the ETL is essentially regarded as an LTI system, the input signals for different target patterns are identical in each method when normalized with the initial and final values of 0.0 and 1.0, respectively. Figure 1(a) shows the normalized input signals in the naïve method and the proposed method with a weight of 1000. For each computed input signal, we measured the temporal patterns of the ETL’s optical power. We confirmed that for all weight values and target patterns, the settling time was lower in the proposed method than in the naïve method. Figure 1(b) shows examples of the results. Plotted are the temporal patterns of the measured optical powers for the six target patterns in both the naïve method and the proposed method with a weight of 1000. The measured optical powers in the figure were obtained by denoising the raw photodiode outputs by a moving mean filter (window size: 7 samples) and linearization (see Linearization in the Methods section). We then identified the time stamps at which the optical powers exceeded (converged within) the initial (final) range to compute the settling times. Figure 1(c) shows the mean and standard deviation of the settling times in each method. The mean settling times in the naïve and the proposed methods differed minimally (by 4.3 ms) when the weight was 0.1, and maximally (by 8.4 ms) when the weight was 10000. In the proposed method, the settling time tended to decrease with increasing weight.

### Focus control for multi-focal imaging

**Figure 2:**
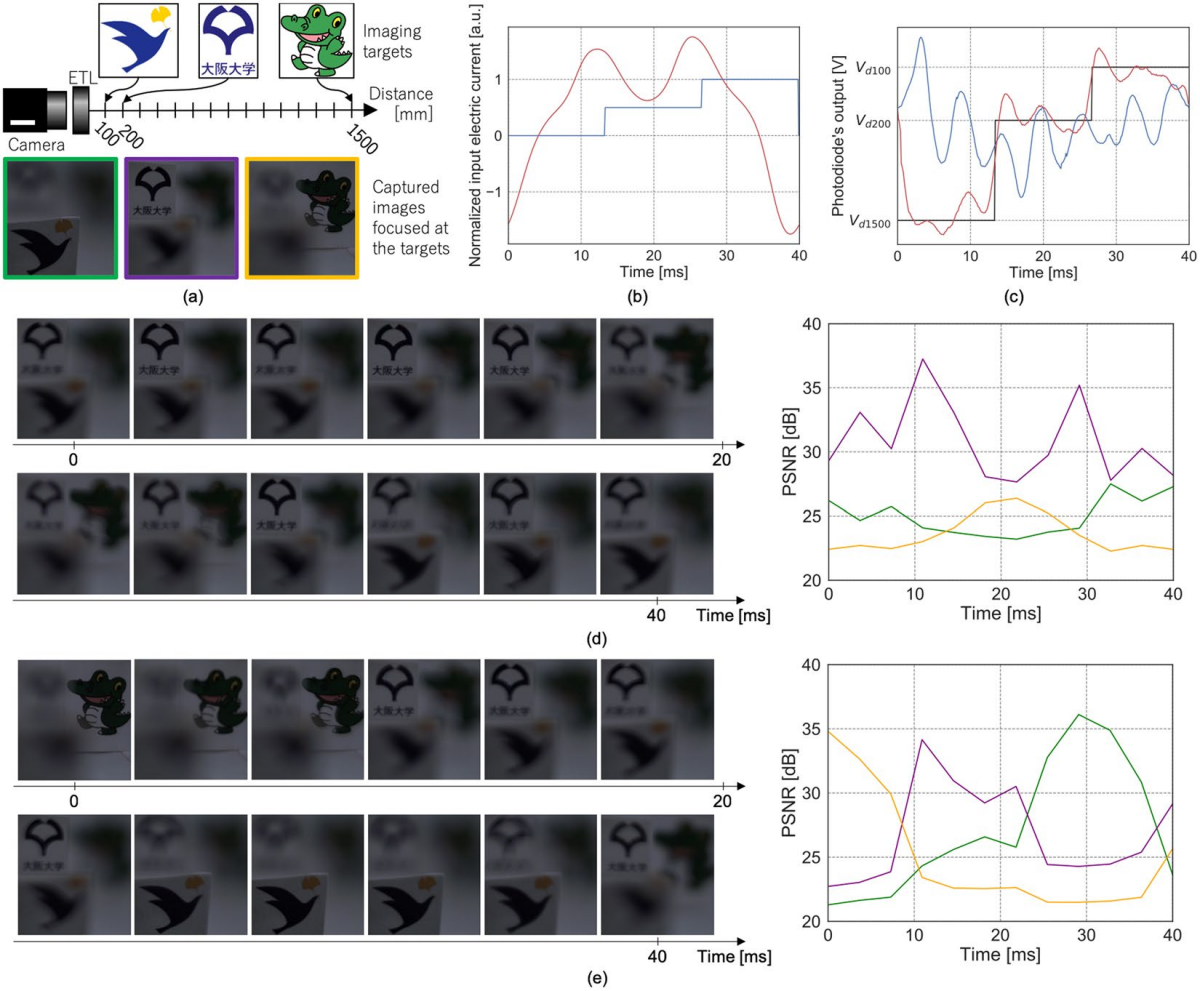
Focus control results in multi-focal imaging. (**a**) Experimental setup. (**b**) Input electric currents. (**c**) Photodiode outputs representing the resulting optical powers and the target pattern, respectively (blue: naïve, red: proposed, black: target). *V*_*d*100_, *V*_*d*200_, and *V*_*d*1500_ represent the photodiode output values when the ETL focused at the targets, respectively. (**d**) Images captured per period in the naïve method (left) and their PSNR values (right), computed by comparing the captured images with those focused at the targets in (**a**). (**e**) As for (**d**), but for the proposed method. The green, purple, and orange plots correspond to the near, middle, and far targets, respectively.

Next, we evaluated the proposed method in an imaging experiment. Three imaging targets were placed at 100, 200, and 1500 mm away from the ETL (Fig. 2(a)). The target temporal pattern of the optical power was a periodic three-step staircase function where each plateau corresponded to each target distance (black line in Fig. 2(c)). Within a period, the ETL was focused from the far to near targets. To check how quickly each imaging target was focused, we need to capture multiple images at each target focusing distance. Considering the frame rate was 300 fps (frames per second) (see Experimental setup section), we determined the period as 40 ms such that four images could be captured for each target focusing distance on average. The input electric current was determined by the naïve and proposed methods. In the naïve method, the input current was a simple staircase function consisting of three plateaus which corresponded to the target plateaus (blue line in Fig. 2(b)). In the proposed method, the input signal was computed by our optimization framework to reproduce the target pattern (red line in Fig. 2(b)). The weight of the smoothing term was 1000.

**Figure 3:**
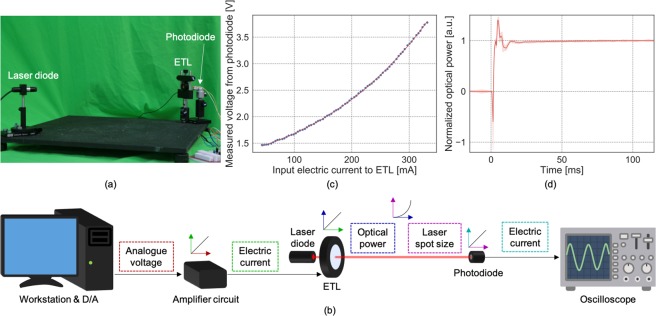
Results of linearization. (**a**) Captured image of the experimental system. (**b**) System overview. (**c**) Measured photodiode voltage versus electric current input to the ETL (blue dots) and its fitted Gaussian function (red line). (**d**) Mean (thick red line) and standard deviation (light red area) of ${\varphi }^{-1}(\bar{y}(t))$.

Figure 2(c) shows the raw outputs of the photodiode, denoised by a moving mean filter with a 7-sample window. The red and blue data represent the resulting output optical powers of the naïve and proposed methods, respectively. The oscillation of the blue line is a consequence of the lens resonance mentioned in Introduction section. On the other hand, the red line for our proposed method can be viewed as an optimized superposition of time-shifted step response that is oscillating as in Fig. 3(d). Thanks to the optimization, the under- and over-shoot of the scaled and time-shifted step response cancel each other, then the original oscillation is successfully suppressed. Figure 2(d,e) show the images captured in each frame of both methods. The proposed method provided sharp appearances of all imaging targets in multiple frames. In contrast, only the imaging target at 200 mm became sharp in the naïve method because the optical power always oscillated around the middle target as can be seen in Fig. 2(c). To evaluate the image qualities, we calculated the peak signal-to-noise ratios (PSNRs) of the captured images, relative to three images focused at the corresponding targets (Fig. 2(a)). For each pair of captured and target images, PSNR was computed as $20{\mathrm{log}}_{10}(255/\sqrt{{\rm{MSE}}})$, where MSE is mean squared error between the images. In the naïve method, PSNRs for the near and far targets were kept low throughout the sequence, while those for the middle target were always high with a certain level of oscillation (Fig. 2(d)). On the other hand, the proposed method successively improved the PSNR for one target after another with less oscillation (Fig. 2(e)). These trends of the image quality are consistent with the temporal patterns of the optical powers in Fig. 2(c).

## Discussion

We proposed a method that optimizes the temporal pattern of the electrical signals input to an ETL, thereby speeding the convergence to the target optical power. The performance of the method was compared with that of naïvely inputting the same waveform as the target pattern. In the first experiment, the proposed method converged the optical power almost twice as fast (on average) as the naïve method. Interestingly, the convergence performance was improved at all weight values, ranging over six orders of magnitude. Therefore, our method does not require tedious fine manual adjustment of the weight. As indicated by the computed input signal in Fig. 1(a), the proposed method can automatically compute a signal with similar properties to the signals in prior works^7,8,24^, which were manually adjusted by low-pass filtering and adding overshoot to the original signal.

In the second experiment, the proposed method was found to improve the captured image qualities in a periodic multi-focal imaging. The proposed method acquired the sharp images of targets set at three distances from the ETL, while the naïve method was unable to focus on the near and far targets. Therefore, our method can potentially improve the image qualities of other imaging and displaying applications. For example, it could acquire more layers in a multi-layer two-photon imaging^3^ at a faster rate than is currently possible. As a display application, our method could realize multi-focal HMDs, reducing the vergence-accommodation conflict, which is currently regarded as one of the most important technical problems in displays, computer graphics, and virtual reality research. Previous works realized multi-focal HMDs by applying ETLs and a special, ultra-high speed projector^23,25^. On the other hand, our method can realize such HMDs with more affordable equipment—more specifically, just by replacing the existing lenses with ETLs. A prior work introduced such a concept^22^, but did not realize it due to the slow response of the ETL. With its faster focus control, our method can potentially do so.

The proposed method is mainly limited by its computational cost. In our current setup, the optimization required approximately 30 seconds. Consequently, our method is currently available only when the sequence of target optical powers is known in advance, and is invalid for applications needing on-demand or closed-loop focus control. In future improvements, the algorithm should faster calculate the input electric current, enabling more interactive applications.

## Methods

### Experimental setup

In the experimental system, the digital signal generated by a workstation (CPU: Intel Xeon E5 v3@3.50 GHz, RAM: 32 GB) was input to a D/A converter (NI USB-6211), and converted to an analog voltage. This voltage was then converted to an electric current by a custom amplifier circuit using an op-amp (LM675T). Finally, the current was fed to an ETL. We installed two ETLs with different aperture sizes: an Optotune EL-10-30-VIS-LD (aperture: 10 mm, optical power: +8.3 to +20 dpt) and an EL-16-40-TC-VIS-20D (aperture: 16 mm, optical power: −10 to +10 dpt). The first experiment used the smaller-aperture lens, as it converges to a target optical power faster than the larger-aperture lens, and we intended to demonstrate accelerated convergence by our proposed method even in a fast ETL. The second experiment used the larger-aperture ETL, which is more suitable for capturing images with a standard industrial camera.

In the first experiment, we measured the optical power of the ETL over time using a photodiode placed 5 mm behind the ETL (see Fig. 3(a)). A laser emitter (wavelength: 635 nm, output power: 0.9 mW) was placed 400 mm in front of the ETL. The laser beam passed through the ETL and intercepted the photodiode. Raising the optical power of the ETL reduced the laser spot size on the photodiode (i.e., increased the laser power density), enhancing the electric current created in the photodiode, from which we indirectly measured the optical power. Considering the ETL response speeds, the sampling rates of the smaller- and larger-aperture ETLs were set to 36 kHz and 10 kHz, respectively. In the second experiment, the images were captured by a high speed camera (Ximea MQ013CG-ON, resolution: 600 × 512 pixels, exposure time: 1 ms, frame rate: 300 fps) (see Fig. 2(a)).

### Modeling and optimization

The system contains an unknown, nonlinear component that transforms the optical power to the beam density on the photodiode (see 3(b)). In order to capture such a nonlinearity, we model the dynamical characteristices from the input electric current *u*(*t*) to output optical power *y*(*t*) by the following *Hammerstein-Wiener model*^28^ with a static nonlinear mapping *ϕ*

1 $$y(t)=\varphi ((u\ast f)(t)),$$

where *f*(*t*) and * are the impulse response of the system and the convolution operator, respectively.

First, in order to identify *ϕ*, constant input currents, which are evenly divided into 75 values ranging from 40 to 330 mA, were injected. According to the manufacturer’s data sheet, this range covers more than 95% of the ETL’s working range. Figure 3(c) plots the output voltages measured by an oscilloscope one second after the application of the input electric currents (the delay avoided undesirable fluctuations of the lens). This voltage-current curve obtained was well fitted by a simple Gaussian function (red line in 3(c)), and was used as *ϕ*.

Next, we identify the impulse response *f*. To this end, the range of input currents (40–220 mA) was divided into seven values. After pairing various two-permutations of the seven currents, we obtained *P*(7, 2) = 42 step functions. Then, the step responses were denoised by a moving mean filter (window size: 7 samples) and globally offset and scaled to match their initial and convergence values to 0 and 1, respectively. These *scaled* step responses are denoted by $\bar{y}(t)$. Note that, if the prototype can be represented by equation (1), ${\varphi }^{-1}(\bar{y}(t))$ should be identical over 42 cases. Figure 3(d) shows the mean and standard deviation of ${\varphi }^{-1}(\bar{y}(t))$. The low standard deviation confirms the nearly identical step responses for the different step signals, verifying the validity of equation (1). It is a well-known fact that the integration of the impulse response is equal to the step function^29^. Then, the derivative of the mean step response (see Fig. 3(d)) was finally used as *f*(*t*). Here we described the linearization process of the smaller-ETL; the larger-aperture ETL was linearized by the same procedure.

The proposed method optimizes the input electric current *u*(*t*) that minimizes the weighted sum of the *L*_1_ norm of the residual error between the temporal patterns of the estimated and target optical powers and the *L*_2_ norm of the second-order differential of the input signal, i.e.,

2 $${u}_{opt}(t)={\rm{\arg }}\,{{\rm{\min }}}_{u(t)}\,\int |{\varphi }^{-1}({y}_{d}(t))-(u\ast f)(t)|+w|u^{\prime\prime} (t){|}^{2}dt,$$

where *y*_*d*_(*t*) and *w* represent the target pattern of the optical power and the weight coefficient, respectively. In r.h.s. of equation (2), $u\ast f=u^{\prime\prime} \ast (f\ast l)$ holds where *l*(*t*) is the lump function *l*(*t*) = *t*^29^. Then, the minimization criterion in the r.h.s. of equation (2) is apparently a convex function of the decision variable *u*″. As the optimization problem is convex, the global optimal solution is guaranteed. The problem was solved by the CVX optimizer in Matlab^30^.

### Accession codes

The datasets generated during and/or analyzed during the current study are available from the corresponding author on reasonable request.

## Supplementary information

Amplifier circuit
