# Supplementary material for: Speeded-Up Focus Control of Electrically Tunable Lens by Sparse Optimization
Source: Sci Rep. 2019 Aug 26;9:12365. doi: 10.1038/s41598-019-48900-z (PMC6710262; doi:10.1038/s41598-019-48900-z)
Supplement: Supplementary file 1 — Amplifier circuit [file 41598_2019_48900_MOESM1_ESM.pdf]

Speeded-Up Focus Control of Electrically Tunable Lens by Sparse Optimization

Daisuke Iwai[1,*,+], Hidetoshi Izawa[1,+], Kenji Kashima[2,+], Tatsuyuki Ueda[1], and Kosuke Sato[1]

[1]Osaka University, Graduate School of Engineering Science, Toyonaka, 560-8531, Japan
[2]Kyoto University, Graduate School of Informatics, Kyoto, 606-8501, Japan
[*]daisuke.iwai@sys.es.osaka-u.ac.jp
[+]these authors contributed equally to this work

Supplementary Material

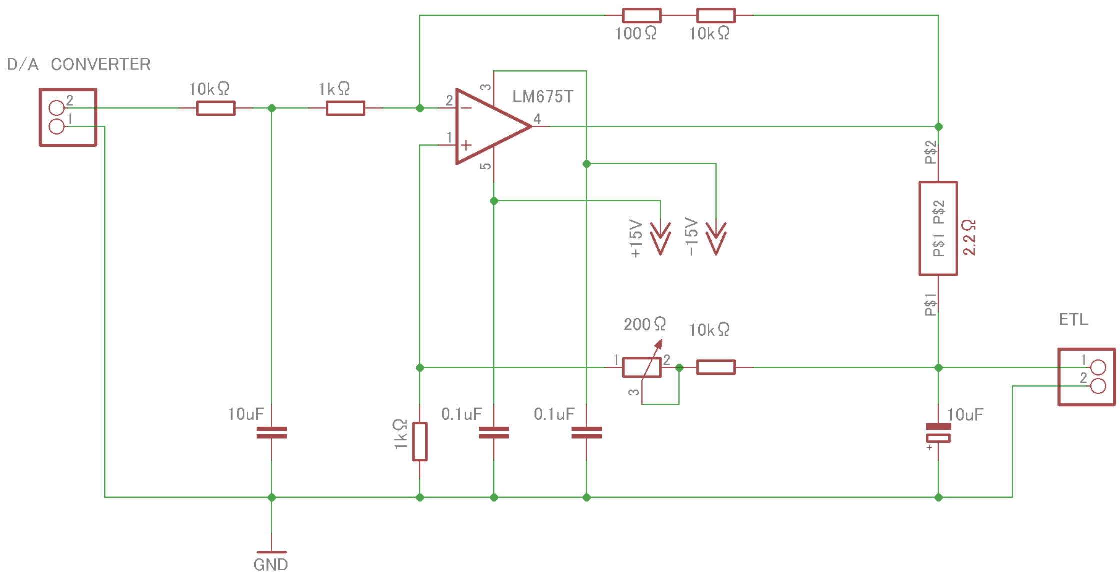

Figure 1. The diagram of the custom amplifier circuit.
